# QTL mapping for grain yield and three yield components in a population derived from two high-yielding spring wheat cultivars

**DOI:** 10.1007/s00122-021-03806-1

**Published:** 2021-03-09

**Authors:** Kyle Isham, Rui Wang, Weidong Zhao, Justin Wheeler, Natalie Klassen, Eduard Akhunov, Jianli Chen

**Affiliations:** 1 Department of Plant Sciences, University of Idaho, Aberdeen, ID USA; 2 Department of Plant Sciences, Kansas State University, Manhattan, KS USA

## Abstract

**Key message:**

Four genomic regions on chromosomes 4A, 6A, 7B, and 7D were discovered, each with multiple tightly linked QTL (QTL clusters) associated with two to three yield components. The 7D QTL cluster was associated with grain yield, fertile spikelet number per spike, thousand kernel weight, and heading date. It was located in the flanking region of FT-D1, a homolog gene of Arabidopsis FLOWERING LOCUS T, a major gene that regulates wheat flowering.

**Abstract:**

Genetic manipulation of yield components is an important approach to increase grain yield in wheat (*Triticum aestivum*). The present study used a mapping population comprised of 181 doubled haploid lines derived from two high-yielding spring wheat cultivars, UI Platinum and LCS Star. The two cultivars and the derived population were assessed for six traits in eight field trials primarily in Idaho in the USA. The six traits were grain yield, fertile spikelet number per spike, productive tiller number per unit area, thousand kernel weight, heading date, and plant height. Quantitative Trait Locus (QTL) analysis of the six traits was conducted using 14,236 single-nucleotide polymorphism (SNP) markers generated from the wheat 90 K SNP and the exome and promoter capture arrays. Of the 19 QTL detected, 14 were clustered in four chromosomal regions on 4A, 6A, 7B and 7D. Each of the four QTL clusters was associated with multiple yield component traits, and these traits were often negatively correlated with one another. As a result, additional QTL dissection studies are needed to optimize trade-offs among yield component traits for specific production environments. Kompetitive allele-specific PCR markers for the four QTL clusters were developed and assessed in an elite spring wheat panel of 170 lines, and eight of the 14 QTL were validated. The two parents contain complementary alleles for the four QTL clusters, suggesting the possibility of improving grain yield via genetic recombination of yield component loci.

**Supplementary information:**

The online version contains supplementary material available at (10.1007/s00122-021-03806-1).

## Introduction

Wheat *(Triticum aestivum,* 2*n* = 6*X* = 42, AABBDD genomes) is one of the most important food crops grown today, as it provides 20% of the calories consumed by the world’s population (Breiman and Graur 1995; FAOSTAT 2017). Wheat yield has continued to increase over time through breeding, but the current rate of increase will be insufficient to meet the future needs of a growing population (Ray et al. 2013). To meet future demand, breeding for increased grain yield must be accelerated. Because grain yield components, such as fertile spikelet number per spike (fSNS), thousand kernel weight (TKW), and productive tiller number per unit area (PTN), typically show higher heritability than grain yield (Wang et al. 2018; Zhang et al. 2018), targeting these components for improvement is an important approach to improve grain yield potential in wheat.

High grain yield is a result of maintaining a good balance of the three key yield component traits in specific environments. Conventional wheat breeding predominately relies on phenotypic selection. Lines must be tested for grain yield in multiple environments across several years to increase the prospect of developing a new, higher yielding cultivar to release. Because of negative correlations among the three yield component traits, simply selecting for improvement in the individual components is ineffective (Wang et al. 2018; Zhang et al. 2018). QTL mapping plays a significant role in increasing grain yield by offering an alternative approach to enhance conventional breeding using molecular markers tightly linked to yield component traits. With the advent of high-density genotyping assays, such as the wheat 90 K (Wang et al. 2014) and 660 K (single-nucleotide polymorphism) SNP arrays (https://wheat.pw.usda.gov/ggpages/topics/Wheat660_SNParraydeveloped_by_CAAS.pdf), significant progress has been recently reported for QTL associated with yield component traits and grain yield. Chen et al. (2020) found a QTL for spikelet number per spike on the short arm of chromosome 7AS that was the pleiotropic effect of *FT-A1*, a gene controlling flowering time. Guan et al. (2020) proposed that *Rht-B1*, a semi-dwarfing gene on chromosome 4B for plant height, is a possible candidate gene for TKW. In addition, using the exome and putative promoter capture assays (Gardiner et al. 2019), Kuzay et al. (2019) and Voss‑Fels et al. (2019) developed a high-resolution genetic map and detected a reliable QTL for spikelet number per spike (SNS) on the long arm of chromosome 7AL and identified a wheat ortholog of the rice aberrant panicle organization 1 (*APO1*) gene as the best candidate gene affecting SNS. Liu et al. (2020) proposed that the *TaFT-D1* may be the candidate gene for two QTL, one related to thousand kernel weight (*QTkw.cas-7D.2*) and another for kernel weight (*Qkw.cas-7D.1*). This progress is a first step toward cloning yield component genes. Although a number of studies examine QTL for multiple yield component traits, which are negatively correlated but work together as a network contributing to grain yield, additional studies are necessary to promote further progress in this area.

The present study used a doubled haploid (DH) mapping population derived from two high-yield spring wheat cultivars with substantial differences in fSNS, PTN, TKW, and HD as measured previously by the corresponding author’s program. The DH mapping population and the two parents were assessed for six traits (GY, fSNS, PTN, TKW, HD, and HT) in eight field trials, two to three traits per year from 2017 to 2019, in Idaho, USA. The mapping population was genotyped with the 90 K SNP Wheat Illumina platform and the exome and promoter capture arrays. This approach enabled simultaneous detection of QTL for GY and the three individual yield components, and results of the study provide a basis for further work to test various marker-assisted selection schemes to improve GY.

## Materials and methods

### Plant materials

Two sets of spring wheat lines were used in the present study. A set of 181 F_1_-derived DH lines from a cross between two high-yielding spring wheat cultivars, ‘UI Platinum’ and ‘LCS Star,’ was used for QTL detection. UI Platinum was developed by the University of Idaho Agricultural Experiment Station in Aberdeen, Idaho, USA, and was released in 2014 (Chen et al. 2016). LCS Star was developed and released by Limagrain Cereal Seeds, USA. Both parents have a semi-dwarfing allele at the *Rht-B1* locus and similar plant height but have alternative alleles for the two major photoperiod response genes. UI Platinum has the photoperiod-insensitive alleles at loci for both *PPD-B1* and *PPD-D1*, while LCS Star has the sensitive alleles. As a result, UI Platinum flowers earlier than LCS Star when grown under short day conditions. The DH lines were created using the wheat by maize hybridization system (Laurie and Bennett 1986) via the services of Heartland Plant Innovation in Kansas, USA.

As a validation panel, a set of 170 spring wheat cultivars and elite lines was used including materials from multiple wheat breeding programs in the Pacific Northwest of the USA and the International Maize and Wheat Improvement Center (CIMMYT) in Mexico. Yield-related traits for this panel were previously published (Wang et al. 2018).

### Phenotypic evaluation and data analysis

The DH mapping population was assessed in four trials under irrigation and in four rainfed trials. The irrigated trials were in Aberdeen, Idaho, USA (42.96° N 112.83° W, elevation 1342 m), in 2017, 2018, and 2019 (17-AB, 18-AB, and 19-AB) and Ashton, Idaho, USA (44.0716° N, 111.4483° W, elevation 1603 m) in 2018 (18-ASH). The rainfed trials were in the relatively high rainfall areas of Moscow, Idaho, USA (46.7324° N, 117.0002° W, elevation 786 m) in 2018 (18-MSC) and Walla Walla, Washington, USA (46.0646° N, 118.3430° W. elevation 287 m) in 2018 (18-WW) and in the dryer area of Soda Springs, Idaho, USA (42.6544° N, 111.6047° W, elevation 1760 m) in 2018 and 2019 (18-SS and 19-SS). The DH and parental lines were arranged in a randomized complete block design with two replications in 18-AB, 19-AB, 18-ASH, 18-SS, 19-SS and with one replication in 17-AB, 18-MSC, and 18-WW. All field trials used standard agronomic practices and seven row plots of 3.0 m by 1.5 m with 0.25 m between rows. Plant growth regulators were not applied, and no severe lodging was observed. The phenotypic data used for the diverse spring wheat panel were sourced from Wang et al. (2018).

Considering the plant growth conditions and labor availability for different trials, the number of traits measured differed among the eight trials. Grain yield in bushels per acre (Bu/A) was assessed in six trials (17-AB, 18-AB, 18-ASH, 18-WW, 18-SS, and 19-SS).

HD was recorded in four trials (18-AB, 18-WW, 19-AB, and 19-SS) and was calculated from January 1 (Julian) to the date when 50% of plants had spikes protruding from flag leaves. Plant height (HT) in inches was recorded in five trials (18-AB, 18-WW, 18-SS, 19-AB, and 19-SS) and was measured from the soil surface to the tip of the spike (awns excluded) at the last stage of maturity before harvest. The fSNS was recorded in seven trials (17-AB, 18-AB, 18-ASH, 18-WW, 18-SS, 19-AB, and 19-SS) and was measured from ten randomly selected spikes that were fully developed before harvest. PTN was recorded in four trials (18-AB, 18-ASH, 18-WW, and 19-AB) and was assessed before harvest as the number of productive tillers per 18 inches in the middle row of each plot. TKW in grams (g) was recorded in seven trials (17-AB, 18-AB, 18-ASH, 18-MSC, 18-SS, 19-AB, and 19-SS) and was assessed by weighing one hundred randomly selected seeds and multiplying the weight by ten to estimate thousand kernel weight. The same phenotyping methods were used for the diverse spring wheat panel. GY, HD, and HT were collected in field trials 16-AB, 17-AB, 17-SS, 18-WW, TKW was collected in trials 16-AB, 17-AB, and 17-SS, and fSNS and PTN were collected in trials 16-AB and 17-AB.

Phenotypic data including BLUP (Best Linear Unbiased Prediction), histograms, correlations, and broad-sense heritability (*H*^2^) were analyzed using JMP Genomics 9.0 (SAS Institute Inc., Cary, NC). The BLUPs across different trials for each trait were calculated considering the genotypes, trials, and replications as random effects in the model. Histograms were tested with the Shapiro–Wilk method and fitted with a normal curve when the p value was > 0.05. Correlation coefficients among different trials for each trait and among different traits were calculated using the “Multivariate” statistical analysis in JMP Genomics 9.0.

In addition, the broad-sense heritability was estimated using the equation *H*^*2*^ = *σ*^2^_*g*_/(*σ*^2^_*g*_ + *σ*^2^_gy_/y + *σ*^2^_gl_/l + *σ*^2^_gyl_/yl + *σ*^2^_*e*_/ylr), where *σ*^2^_*g*_ is the variance of genotypes, *σ*^2^_gy_ is the variance of genotype-year, *σ*^2^_gl_ is the variance of genotype-location, *σ*^2^_gyl_ is the variance of genotype-location-year, *σ*^2^_*e*_ is the residual variance, *e* is the total trial numbers, and *r* is the number of replications in each trial (Fehr 1987).

### Genetic map construction and linkage analysis

The DH and parental lines were genotyped at the USDA-ARS Small Grains Genotyping Laboratory in Fargo, ND, USA, using the Infinium wheat SNP 90 K iSelect assay (Illumina Inc., San Diego, CA, USA) developed by the International Wheat SNP Consortium (Wang et al. 2014). This assay yielded 81,587 SNPs and was used in the linkage analysis in JMP Genomics 9.0. First, the linkage groups were determined with automated hierarchical and K-means clustering to remove the colocated markers in the linkage group. Then, the markers within each single linkage group were ordered using the cM Kosambi mapping function and the accelerated map order optimization algorithm in the software’s ‘linkage map order' function. Linkage groups were separated when the genetic distance between bordering markers was greater than 50 cM.

### QTL analysis

QTL analysis was conducted using individual and BLUP datasets for GY, fSNS, TKW, PTN, HD, and HT by the composite interval mapping (CIM) method in JMP Genomics 9.0. Significant QTL were determined with the expectation maximization algorithm at a threshold of 2.5 (LOD > 2.5). The software output provided a proportion of phenotypic variance (*R*^*2*^) and the additive effects for each marker. The source of the allelic effect of the parent UI Platinum or LCS Star was indicated by negative or positive estimates of the additive effects, respectively. To investigate the QTL × QTL interaction effects for a specific trait, multiple interval mapping was conducted using BLUP datasets in JMP Genomics 9.0 with the Haley–Knott Regression algorithm. The logarithm of the odds (LOD) threshold of 2.5 was set for entry and retention in the model. In addition, to dissect the confounding effects of heading date on yield, QTL analyses were conducted for all grain yield datasets using composite interval mapping with heading date (BLUP dataset) as a cofactor variable.

To determine the physical positions for identified QTL regions, a BLAST search (https://urgi.versailles.inra.fr/blast_iwgsc/?dbgroup=wheat_iwgsc_refseq_v1_chromosomes&program=blastn) was preformed to align the QTL-associated peak and flanking SNP marker sequences with the reference wheat genome assembly constructed in the cv. Chinese Spring sequence (RefSeq v1.0, the International Wheat Genome Consortium).

### KASP marker development

To saturate the identified major QTL, additional SNPs between the two parents in the target QTL regions were identified based on the exome and putative promoter capture data (Gardiner et al. 2019) obtained from the Triticeae Toolbox (T3) (https://triticeaetoolbox.org/wheat/). These SNPs were genotyped using KBioscience’s Competitive Allele-Specific PCR (KASP). The primers for KASP markers were designed based on each identified SNP using PolyMarker (Ramirez-Gonzalez et al. 2015). KASP primers were verified on the parents and then used to screen the DH population. The KASP assays were performed in a CFX384 Touch™ Real-Time PCR Detection System (Bio-Rad, Hercules, CA). The reaction system and PCR conditions were based on the protocol from LGC Genomics. The plate was read and set at 25 ºC for the last step, and the data were visualized and analyzed using the allelic discrimination function in CFX Maestro software (Bio-Rad, Hercules, CA).

### QTL validation in a diverse spring wheat line panel

Peak SNPs for major QTL identified in the DH mapping population were converted to KASP markers and were genotyped in a spring wheat panel of 170 diverse lines, which were developed by the University of Idaho, Washington State University, University of California, Davis, Montana State University, and CIMMYT described in Wang et al. (2017). Allelic effect of a QTL on GY, fSNS, PTN, and TKW was analyzed with a t test in JMP Genomics 9.0.

## Results

### Phenotypic analysis of GY HD, HT, fSNS, TKW, and PTN

**Fig. 1:**
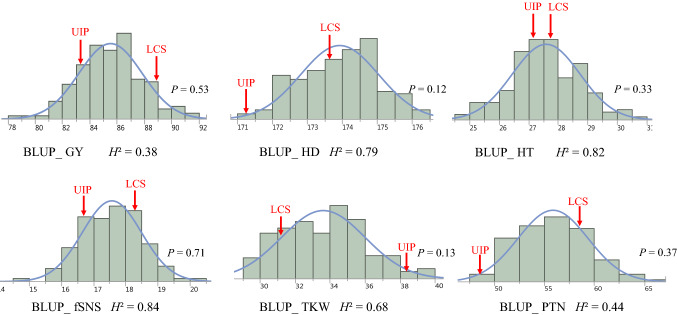
Distribution of the BLUP data for GY, HD, HT, fSNS, TKW, and PTN in the DH population. The BLUP values of the parents are indicated on the histogram plots using the red arrows. The broad-sense heritability for each trait is shown below each histogram

Based on the BLUP data, the two parents differed significantly in five out of the six traits with the exception being HT (Fig. 1). LCS Star had higher trait values for GY, HD, fSNS, and PTN, while UI Platinum had higher trait values in TKW. BLUP datasets of all traits for the DH population showed normal distributions with *P* values from 0.12 to 0.71 using the Shapiro–Wilk method in JMP Genomics 9.0, which suggests polygenic inheritance of these traits (Fig. 1). Transgressive segregation was common at both ends of the distribution for GY, fSNS, and HT, and at one end of the distribution for TKW, PTN, and HD (Fig. 1). UI Platinum had the greatest TKW values, shortest HD, and the least PTN (Fig. 1). The HD, HT, fSNS, and TKW showed high broad-sense heritability at 0.79, 0.82, 0.84, and 0.68, respectively, suggesting a strong genetic contribution to these traits in the population. GY and PTN showed moderate broad-sense heritability of 0.38 and 0.44, respectively, indicating these traits were more affected by environmental factors. Consistent with the heritability values, the correlation coefficients (*r*^*2*^) among different trials for HD and HT ranged from 0.6 to 0.9, and for fSNS and TKW from 0.3 to 0.7. The *r*^*2*^ among different trials for GY and PTN ranged from 0.2 to 0.4 (Table 1).

**Table 1:** Correlations among different trials for each trait and correlations among all traits based on Best Linear Unbiased Prediction (BLUP) values in the UI Platinum x LCS Star derived doubled haploid population

Grain yield						
Trial	17-AB	18-AB	18-ASH	18-WW	18-SS	19-SS
18-AB	0.30***					
18-ASH	0.37***	0.17*				
18-WW	0.49***	0.34***	0.47***			
18-SS	0.26**	0.06	0.12	0.38***		
19-SS	0.27***	0.19*	0.16*	0.39***	0.24**	
BLUP	0.74***	0.43***	0.57***	0.54***	0.26**	0.37***

Heading date				
Trial	18-AB	18-WW	19-AB	19-SS
18-WW	0.83***			
19-AB	0.87***	0.82***		
19-SS	0.85***	0.83***	0.93***	
BLUP	0.85***	0.83***	0.86***	0.85***

Height					
Trial	18-AB	18-WW	18-SS	19-AB	19-SS
18-WW	0.81***				
18-SS	0.60***	0.63***			
19-AB	0.82***	0.77***	0.59***		
19-SS	0.48***	0.74***	0.66***	0.68***	
BLUP	0.88***	0.85***	0.75***	0.82***	0.80***

Fertile spikelet number per spike							
Trial	17-AB	18-AB	18-ASH	18-WW	18-SS	19-AB	19-SS
18-AB	0.59***						
18-ASH	0.53***	0.69***					
18-WW	0.48***	0.69***	0.59***				
18-SS	0.49***	0.69***	0.60***	0.53***			
19-AB	0.57***	0.83***	0.66***	0.63***	0.66***		
19-SS	0.55***	0.81***	0.68***	0.63***	0.69***	0.77***	
BLUP	0.59***	0.89***	0.86***	0.79***	0.85***	0.81***	0.89***

Thousand kernel weight							
Trial	17-AB	18-AB	18-ASH	18-MSC	18-SS	19-AB	19-SS
18-AB	0.43***						
18-ASH	0.41***	0.33***					
18-MSC	0.29**	0.45***	0.37***				
18-SS	0.34***	0.31***	0.28**	0.24**			
19-AB	0.40***	0.54***	0.33***	0.44***	0.19**		
19-SS	0.45***	0.34***	0.45***	0.30***	0.53***	0.21**	
BLUP	0.70***	0.75***	0.68***	0.65***	0.60***	0.72***	0.63***

Productive tiller number per unit area				
Trial	18-AB	18-ASH	18-WW	19-AB
18-ASH	0.39***			
18-WW	0.40***	0.41***		
19-AB	0.43***	0.25**	0.34***	
BLUP	0.70***	0.64***	0.60***	0.50***

BLUP value					
Trait	GY	HD	HT	fSNS	TKW
HD	0.30***				
HT	0.27***	0.25***			
fSNS	0.37***	0.74***	0.20**		
TKW	− 0.04	− 0.38***	0.21**	-0.33***	
PTN	− 0.02	− 0.12	− 0.22**	− 0.19**	− 0.49***

Significance level: ***, **, and * indicate *p* < 0.001, 0.01, and 0.05, respectively

Correlations among the different traits showed that GY positively correlated with fSNS, HD, and HT, but there was no significant correlation between GY, TKW and PTN. The fSNS positively correlated with GY, HD, and HT but negatively correlated with PTN and TKW. TKW negatively correlated with PTN, fSNS, HD, but positively correlated with HT. PTN negatively correlated with TKW, fSNS, and HT but did not significantly correlate with GY and HD (Table 1).

### Linkage group construction and marker analysis

Of the 81,587 SNPs called from the 90 K iSelect SNP array, 14,236 were polymorphic between UI Platinum and LCS Star. After excluding the markers that either cosegregated at the same position or with missing values in more than 10% of the lines, a total of 1276 SNP markers remained for linkage map construction. A total of 48 linkage groups were constructed, representing all 21 hexaploid wheat chromosomes. Chromosomes 6A, 2B, and 1D were represented by one linkage group each; chromosomes 1A, 2A, 4A, 3B, 4B, 5B, 6B, 2D, 4D, and 7D were represented by two linkage groups each. Chromosomes 3A, 5A, 1B, 7B, 3D, 5D, and 6D were represented by three linkage groups each, and chromosomes 7A was represented by four linkage groups (Supplemental Table 1). The total length of the linkage map was 3892.81 cM, with a mean marker density of 0.33 marker per cM. The map of the A genome had 489 markers (38%) with a total length of 1322.46 cM and an average marker density of 0.37 marker per cM. The map of the B genome had 583 markers (46%) with a total length of 1546.01 cM and an average marker density of 0.38 marker per cM. The map of the D genome included 204 markers (16%) with a total length of 1024.34 cM and an average marker density of 0.20 markers per cM. The D genome had the lowest marker coverage, especially for chromosomes 2D, 4D, 5D, and 7D, and the marker density for these chromosomes was less than 0.20 markers per cM (Supplemental Table 1).

### QTL detection

#### QTL for GY

**Fig. 2:**
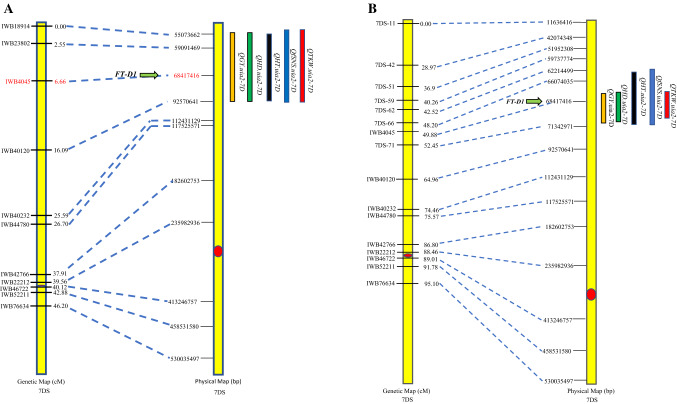
Genetic and physical positions for QTL clusters on chromosome 7D based on the 90 K SNP map (**a**) and the saturated map with additional KASP markers (**b**). Collinearity relationships among the genetic map from the present study and the physical map from RefSeq v1.0 for the identified QTL clusters were indicated by dash lines. The QTL for GY, HD, HT, fSNS, and TKW were detected based on BLUP datasets and were indicated by orange, green, black, blue, and red bars, respectively

Only one QTL for GY, *QGy.uia2-7D*, was detected on chromosome 7D in four of seven datasets (17-AB, 18-ASH, 18-WW, and BLUP data) (Fig. 2a and Table 2). *QGy.uia2-7D* explained 14 to 17% of the phenotypic variation, and its peak marker was mapped in the position of the *FT-D1*, a homolog gene of Arabidopsis *FLOWERING LOCUS T*, a major gene that regulates wheat flowering (Yan et al. 2006; Chen et al. 2014). An LCS Star allele for this QTL contributed to high GY. When heading date (BLUP dataset) was used as a cofactor variable in the QTL analysis, this QTL remained significant in three datasets (17-AB, 18-WW, and BLUP data) (Table 2).

**Table 2:** Quantitative trait loci (QTL) detected for grain yield (GY), heading date (HD), plant height (HT), fertile spikelet number per spike (fSNS), thousand kernel weight (TKW), and productive tiller number (PTN) in the UI Platinum x LCS Star-derived doubled haploid population

Trait	QTL	Environment	Interval	Positions (cM)	Peak Marker	Peak Position (cM)	Physical Position (bp)	LOD	Effect^a^	*R*^*2*^ (%)
GY	*QGY.uia2-7D*	17-AB	*IWB18914-IWB42766*	0.00–37.91	*IWB4045*	6.66	68,417,416	6.88	19.59	16
		18-ASH	*IWB23802-IWB40232*	0.55–25.59	*IWB4045*	6.66	68,417,416	5.74	9.25	14
		18-WW	*IWB23820-IWB40120*	0.55–24.09	*IWB4045*	6.66	68,417,416	7.13	6.60	17
		BLUP	*IWB23802-IWB44453*	0.00–24.09	*IWB4045*	6.66	68,417,416	7.14	1.89	17
	*HD as covariate:* *QGY.uia2-7D*	17-AB	*IWB23820-IWB40120*	0.55–24.09	*IWB4045*	6.66	68,417,416	4.00	17.55	10
		18-WW	*IWB4045-IWB40120*	6.66–24.09	*IWB4045*	6.66	68,417,416	4.31	6.03	10
		BLUP	*IWB23802-IWB4045*	0.55–6.66	*IWB4045*	6.66	68,417,416	3.27	1.65	8
	*KASP Markers: QGY.uia2-7D*	17-AB	*IWB4045-IWB4045*	55.45–55.45	*IWB4045*	57.45	68,417,416	6.88	19.59	16
		18-ASH	*Kasp62215-Kasp71343*	42.52–53.88	*7DS-71*	49.88	71,343,000	6.31	9.80	15
		18-WW	*Kasp66074-IWB4045*	48.20–55.45	*IWB4045*	57.45	68,417,416	5.29	9.75	13
		19-SS	*Kasp66074-Kasp66074*	48.20–48.20	*7DS-66*	48.2	66,074,000	4.61	9.28	11
		BLUP	*Kasp71343-IWB4045*	49.88–55.45	*IWB4045*	57.45	68,417,416	2.74	2.24	7
HD	*QHD.uia2-4B*	18-AB	*IWB73001-IWB13349*	45.70–69.19	*IWB23968*	64.21	518,682,966	4.31	− 1.46	10
		18-WW	*IWB73001-IWB23337*	45.70–74.29	*IWB64397*	62.56	517,623,727	4.70	− 1.27	11
		19-AB	*IWB23968-IWB23968*	64.21–64.21	*IWB23968*	64.21	518,682,966	2.65	− 0.95	7
		BLUP	*IWB73001-IWB13349*	45.70–69.19	*IWB23968*	64.21	518,682,966	3.17	− 0.52	8
	*QHD.uia2-6A*	18-AB	*IWB3945-IWB10738*	6.08–34.83	*IWB9036*	12.16	19,242,465	4.59	− 1.38	11
		18-WW	*IWB3945-IWB45465*	6.08–44.81	*IWB11102*	14.37	61,024,039	4.77	− 1.22	11
		19-AB	*IWB11102-IWB10738*	14.37–34.83	*IWB76736*	23.76	NA^b^	2.69	− 0.91	7
		19-SS	*IWB39323-IWB34744*	6.63–55.35	*IWB11102*	14.37	61,024,039	5.25	− 1.17	13
		BLUP	*IWB3945-IWB2006*	6.08–49.80	*IWB11102*	14.37	61,024,039	2.98	− 0.46	7
	*QHD.uia2-7B*	18-AB	*IWB3325-IWB76084*	13.84–22.17	*IWB76332*	22.06	5,922,944	4.21	1.35	10
		18-WW	*IWB10879-IWB76332*	11.08–22.06	*IWB76332*	22.06	5,922,944	5.89	1.44	14
		19-AB	*IWB10879-IWB76084*	11.08–22.06	*IWB76332*	22.06	5,922,944	5.70	1.45	14
		19-SS	*IWB76332-IWB76084*	16.06–22.17	*IWB76332*	22.06	5,922,944	2.85	0.89	7
		BLUP	*IWB10879-IWB76084*	11.08–22.17	*IWB76332*	22.06	5,922,944	8.59	0.79	20
	*QHD.uia2-7D*	18-AB	*IWB18914-IWB40120*	0.00–24.09	*IWB4045*	6.66	68,417,416	27.48	3.77	50
		18-WW	*IWB18914-IWB40120*	0.00–24.09	*IWB4045*	6.66	68,417,416	13.55	2.23	29
		19-AB	*IWB18914-IWB40120*	0.00–24.09	*IWB4045*	6.66	68,417,416	13.14	2.27	29
		19-SS	*IWB18914-IWB40120*	0.00–24.09	*IWB4045*	6.66	68,417,416	13.51	2.01	29
		BLUP	*IWB18914-IWB40120*	0.00–24.09	*IWB4045*	6.66	68,417,416	26.60	1.40	49
	*KASP Markers: QHD.uia2-7D*	18-AB	*Kasp62215-IWB4045*	42.52–63.45	*7DS-71*	51.88	71,343,000	19.74	4.61	39
		18-WW	*Kasp62215-IWB4045*	42.52–63.45	*7DS-71*	53.88	71,343,000	7.06	2.65	16
		19-AB	*Kasp62215-IWB4045*	42.52–63.45	*7DS-71*	51.88	71,343,000	14.47	2.54	31
		19-SS	*Kasp62215-IWB4045*	42.52–63.45	*IWB4045*	48.2	68,417,416	12.71	2.69	28
		BLUP	*Kasp62215-IWB4045*	42.52–63.45	*IWB4045*	48.2	68,417,416	21.40	1.69	42
HT	*QHT.uia2.4A-1*	18-AB	*IWB11778-IWB18325*	5.53–24.33	*IWB33052*	20.46	102,614,749	11.71	2.22	26
		18-WW	*IWB-11778-IWB18325*	5.53–24.33	*IWB33052*	20.46	102,614,749	9.16	5.33	21
		19-AB	*IWB34660-IWB31143*	0.00–16.04	*IWB65970*	9.19	596,836,024	5.19	1.35	12
		18-SS	*IWB65970-IWB33052*	7.19–20.46	*IWB31143*	16.04	38,447,212	3.04	0.93	7
		BLUP	*IWB-12303-IWB18325*	4.21–32.33	*IWB33052*	20.46	102,614,749	8.81	0.87	20
	*QHT.uia2-4A-2*	18-AB	*IWB60934-IWB12351*	37.30–59.53	*IWB11801*	57.1	713,120,773	5.30	− 1.49	13
		18-WW	*IWB5681-IWB12351*	50.68–59.53	*IWB12351*	59.53	715,512,708	3.95	− 3.38	10
		19-AB	*IWB11801-IWB12351*	57.10–60.09	*IWB1056*	59.53	715,512,708	4.35	− 1.23	10
		18-SS	*IWB20951-IWB1375*	22.28–36.63	*IWB20951*	24.28	618,040,255	3.97	− 1.07	10
		19-SS	*IWB37469-IWB19112*	30.08–47.09	*IWB1375*	34.63	689,853,869	3.73	− 0.93	9
		BLUP	*IWB37469-IWB19112*	30.08–47.09	*IWB25876*	41.18	663,273,738	5.04	− 0.71	12
	*QHT.uia2.5A*	18-AB	*IWB24570-IWB24570*	60.58–64.58	*IWB24570*	60.58	NA	3.69	− 0.94	9
		18-SS	*IWB64718-IWB38700*	29.57–51.17	*IWB3232*	40.11	665,471,359	2.69	− 1.08	7
		BLUP	*IWB15328-IWB38700*	16.75–64.58	*IWB10313*	43.42	673,549,466	2.60	− 0.47	6
	*QHT.uia2-5D*	18-AB	*IWB6557-IWB6557*	82.37–106.37	*IWB6557*	84.37	107,583,992	5.23	3.50	12
		18-WW	*IWB6557-IWB6557*	82.37–106.37	*IWB6557*	84.37	107,583,992	2.64	6.90	7
		BLUP	*IWB6557-IWB6557*	82.37–106.37	*IWB6557*	82.37	107,583,992	2.83	1.19	7
	*QHT.uia2-7D*	18-AB	*IWB23802-IWB40120*	0.55–16.09	*IWB4045*	6.66	68,417,416	10.50	2.16	23
		18-WW	*IWB23802-IWB40120*	0.55–16.09	*IWB44453*	12.77	NA	6.36	4.45	15
		19-AB	*IWB23802-IWB40120*	0.55–22.09	*IWB4045*	8.66	68,417,416	6.35	1.45	15
		BLUP	*IWB23802-IWB40120*	0.55–24.09	*IWB44453*	12.77	NA	11.06	1.00	25
	*KASP Markers: QHT.uia2-7D*	18-AB	*Kasp51953-IWB4045*	36.90–63.45	*7DS-66*	48.2	66,074,000	4.99	1.95	12
		18-WW	*Kasp71343-IWB4045*	49.88–63.45	*IWB4045*	57.45	68,417,416	5.56	1.81	13
		19-AB	*Kasp62215-IWB4045*	42.52–63.45	*IWB4045*	57.45	68,417,416	8.14	2.43	19
		BLUP	*Kasp62215-Kasp62215*	45.52–46.52	*7DS-62*	46.52	62,215,000	4.09	0.94	10
fSNS	*QfSNS.uia2-5D*	17-AB	*IWB7620-IWB3446*	3.88–24.01	*IWB17912*	18.43	464,633,623	3.51	0.80	9
		18-AB	*IWB7620-IWB49479*	3.88–38.12	*IWB17912*	22.43	464,633,623	4.81	0.63	12
		18-SS	*IWB7620-IWB34466*	3.88–28.01	*IWB17912*	20.43	464,633,623	4.65	0.91	11
		19-SS	*IWB7620-IWB49479*	13.88–38.12	*IWB34466*	24.01	586,352,242	5.56	0.68	13
		BLUP	*IWB7620-IWB34466*	7.88–28.01	*IWB17912*	22.43	464,633,623	7.56	0.54	18
	*QfSNS.uia2-6A*	17-AB	*IWB10644-IWB45465*	13.82–44.81	*IWB10738*	34.83	535,894,664	3.68	− 0.855	9
		18-AB	*IWB39323-IWB2006*	6.63–49.80	*IWB63176*	16.92	63,562,964	4.89	− 0.66	12
		18-WW	*IWB-63176-IWB10738*	16.92–36.83	*IWB10321*	32.62	288,411,565	3.17	− 0.50	8
		18-ASH	*IWB10738-IWB45465*	34.83–46.81	*IWB45465*	44.81	646,630,596	3.97	− 0.97	10
		19-AB	*IWB10644-IWB34744*	13.82–55.35	*IWB76733*	33.17	454,649,338	5.13	− 0.75	12
		18-SS	*IWB35333-IWB2006*	33.17–49.80	*IWB45465*	44.81	646,630,596	4.21	− 0.85	10
		19-SS	*IWB35333-IWB45465*	33.17–46.81	*IWB35333*	23.76	NA	4.75	− 0.66	11
		BLUP	*IWB35333-IWB2006*	33.17–53.80	*IWB45465*	44.81	646,630,596	8.55	− 0.56	20
	*QfSNS.uia2-7B*	18-WW	*IWB10879-IWB76332*	11.08–22.06	*IWB76332*	16.06	5,922,944	9.33	0.94	21
		18-ASH	*IWB10879-IWB76332*	11.08–22.06	*IWB76332*	16.06	5,922,944	5.81	1.20	14
		19-AB	*IWB76332-IWB76332*	16.06–20.06	*IWB76332*	16.06	5,922,944	4.52	0.71	11
		19-SS	*IWB10879-IWB76332*	11.08–22.06	*IWB76332*	16.06	5,922,944	5.27	0.69	13
		BLUP	*IWB10879-IWB76332*	11.08–22.06	*IWB76332*	16.06	5,922,944	10.72	0.66	24
	*QfSNS.uia2-7D*	17-AB	*IWB18914-IWB40120*	0.55–24.09	*IWB4045*	6.66	68,417,514	6.58	1.17	15
		18-AB	*IWB18914-IWB40120*	0.00–24.09	*IWB4045*	6.66	68,417,514	21.74	1.59	42
		18-WW	*IWB18914-IWB40120*	0.00–24.09	*IWB4045*	6.66	68,417,514	7.19	0.83	17
		18-ASH	*IWB18914-IWB40120*	0.00–24.09	*IWB4045*	6.66	68,417,514	7.66	1.31	17
		19-AB	*IWB18914-IWB40120*	0.00–24.09	*IWB4045*	6.66	68,417,514	22.50	1.80	44
		18-SS	*IWB18914-IWB40120*	0.00–24.09	*IWB4045*	6.66	68,417,514	12.55	1.59	27
		19-SS	*IWB18914-IWB40120*	0.00–24.09	*IWB4045*	6.66	68,417,514	14.50	1.25	31
		BLUP	*IWB18914-IWB40120*	0.00–24.09	*IWB4045*	6.66	68,417,514	22.30	1.04	43
	*KASP Markers: QfSNS.uia2-7D*	17-AB	*Kasp62215-Kasp71343*	42.52–53.88	*7DS-62*	46.52	62,115,000	6.03	1.50	15
		18-AB	*Kasp62215-IWB4045*	42.52–59.45	*7DS-71*	53.88	71,343,000	9.63	1.72	22
		18-ASH	*Kasp62215-Kasp66074*	42.52–48.20	*IWB4045*	48.2	68,417,514	5.01	1.47	12
		18-WW	*Kasp62215-Kasp66074*	42.52–48.20	*IWB4045*	48.2	68,417,514	8.03	1.67	14
		19-AB	*Kasp51953-IWB40120*	36.9–66.96	*IWB4045*	48.2	68,417,514	19.40	2.05	39
		18-SS	*Kasp62215-Kasp66074*	42.52–48.2	*7DS-62*	46.52	66,074,000	8.00	1.51	18
		19-SS	*Kasp62215-IWB4045*	42.52–63.45	*7DS-71*	51.88	71,343,000	10.91	1.37	24
		BLUP	*Kasp62215-IWB4045*	42.52–63.45	*7DS-71*	53.88	71,343,000	15.81	1.16	33
TKW	*QTKW.uia2-4A*	17-AB	*IWB23168-IWB18325*	18.8–28.33	*IWB18325*	24.33	394,968,199	3.12	1.72	8
		18-AB	*IWB31143-IWB18325*	16.04–34.33	*IWB18325*	24.33	394,968,199	5.30	2.52	13
		18-MSC	*IWB18250-IWB20649*	14.17–22.67	*IWB31143*	16.04	38,447,212	3.36	1.75	8
		18-SS	*IWB18325-IWB35445*	24.33–59.34	*IWB35445*	43.34	21,063,715	4.02	1.77	10
		BLUP	*IWB65970-IWB18325*	11.19–32.33	*IWB46934*	19.91	488,252,088	5.31	1.69	13
		17-AB	*IWB10644-IWB38557*	13.82–27.42	*IWB12868*	17.13	73,723,540	4.61	− 2.20	11
	*QTKW.uia2-6A*	18-SS	*IWB12868-IWB1754*	17.13–39.6	*IWB38557*	27.42	201,348,573	6.14	− 2.12	14
		BLUP	*IWB34488-IWB1754*	19.9–39.6	*IWB38557*	25.42	201,348,573	4.53	− 1.51	11
	*QTKW.uia2-7D*	18-AB	*IWB18914-IWB40120*	0.00–24.09	*IWB4045*	6.66	68,417,514	12.12	− 4.28	27
		19-AB	*IWB18914-IWB40120*	0.00–22.09	*IWB4045*	6.66	68,417,514	8.25	− 3.38	19
		BLUP	*IWB18914-IWB40120*	0.00–22.09	*IWB4045*	6.66	68,417,514	7.30	− 1.80	17
	*KASP Markers: QTKW.uia2-7D*	18-AB	*Kasp51953-IWB4045*	36.9–63.45	*7DS-62*	46.52	62,215,000	12.45	− 5.33	27
		18-SS	*IWB40120-IWB40232*	68.96–74.46	*IWB40120*	72.96	92,570,641	2.68	− 2.18	7
		19-AB	*Kasp62215-IWB4045*	42.52–59.45	*7DS-62*	46.52	62,215,000	5.14	− 4.11	12
		BLUP	*Kasp62215-IWB40120*	42.52–72.96	*IWB4045*	55.45	68,417,514	5.49	− 2.12	13
PTN	*QPTN.uia2-4A*	18-AB	*IWB11778-IWB-18325*	5.53–24.33	*IWB31143*	16.04	38,447,212	3.67	− 4.42	9
		18-ASH	*IWB54290-IWB35445*	40.99–47.34	*IWB77282*	42.78	24,755,594	2.65	− 2.99	7
		BLUP	*IWB18250-IWB18325*	14.17–24.33	*IWB33052*	20.46	102,614,749	3.95	− 1.99	10
	*QPTN.uia2-6A*	18-AB	*IWB34957-IWB10321*	10.5–32.62	*IWB38557*	25.42	201,348,573	8.96	6.93	20
		18-ASH	*IWB9036-IWB10321*	12.16–32.62	*IWB68246*	22.11	410,915,261	4.43	3.91	11
		18-WW	*IWB10321-IWB-11269*	32.62–41.47	*IWB35333*	33.17	454,649,338	3.02	3.57	7
		19-AB	*IWB34957-IWB10321*	10.5–29.30	*IWB68246*	22.11	410,915,261	7.60	5.31	18
		BLUP	*IWB34957-IWB10321*	10.5–32.62	*IWB38557*	25.42	201,348,573	8.70	3.04	20

^a^The contribution of the higher phenotype value from either UIP or LCS is indicated by negative or positive number, respectively. ^b^NA indicates the physical position of that marker cannot be determined through the BLAST search that used in the present study

#### QTL for HD

Four QTL were detected for HD (Table 2), one each on chromosomes 4B, 6A, 7B, and 7D. *QHd.uia2-7D* was detected in all five datasets (18-AB, 18-WW, 19-AB, 19-SS, and the BLUP data) and had the largest effect among the four QTL, explaining 29 to 50% of phenotypic variation (Table 2). Like GY, this QTL was mapped in the flanking region of *FT-D1* (Fig. 2a). *QHd.uia2-7B* was also detected in all five datasets (18-AB, 18-WW, 19-AB, 19-SS, and the BLUP data) and explained less phenotypic variation (20% based on BLUP data) than *QHd.uia2-7D*, but more than both QTL *QHd.uia2-4B* and *QHd.uia2-6A*. *QHd.uia2-4B* and *QHd.uia2-6A* only explained 8 and 7% of overall phenotypic variation, respectively, based on the BLUP values. LCS Star alleles for *QHd.uia2-7D* and *QHd.uia2-7B* contributed to late heading, while LCS Star alleles for *QHd.uia2-4B* and *QHd.uia2-6A* contributed to early heading.

#### QTL for HT

Five QTL were detected for HT, two on chromosome 4A, and one each on 5A, 5D, and 7D (Table 2). *QHt.uia2-4A.1* was detected in five of six datasets (18-AB, 18-WW, 18-SS, 19-AB, and BLUP data), explaining 7 to 26% of the phenotypic variation (Table 2). *QHt.uia2-4A.2* was detected in all datasets (18-WW, 18-SS, 18-AB, 19-AB, 19-SS, and the BLUP data), explaining 9 to 13% of the phenotypic variation. *QHt.uia2-5A* was detected on the long arm of chromosome 5A in three of six datasets (18-AB, 18-SS, and BLUP data), explaining 6 to 9% of the phenotypic variation. *QHt.uia2-5D* was detected on the short arm of chromosome 5D in three of six datasets (18-WW, 18-AB, and BLUP data), explaining 7 to 12% of the phenotypic variation. *QHt.uia2-7D* was detected in four of six datasets (18-AB, 18-WW, 19-AB, and BLUP data), explaining 15 to 25% of the phenotypic variation. Among these QTL, increased plant height was contributed by the LCS Star alleles for *QHt.uia2-4A.1*, *QHt.uia2-5D*, and *QHt.uia2-7D* and by the UI Platinum allele for *QHt.uia2-4A.2* and *QHt.uia2-5A*. Like GY and HD, the QTL *QHt.uia2-7D* had the biggest effect on HT and was mapped in the flanking region of *FT-D1* (Table 2 and Fig. 2a).

#### QTL for fSNS

Four QTL were detected for fSNS (Table 2) on chromosomes 5D, 6A, 7B and 7D. *QfSns.uia2-5D* was detected in five of eight datasets (17-AB, 18-AB, 18-SS, 19-SS, and BLUP data) and explained 9 to 18% of the phenotypic variation (Table 2). *QfSns.uia2-7B* was detected in five of eight datasets (18-WW, 18-ASH, 19-AB, 19-SS, and BLUP data) and explained 11 to 24% of the phenotypic variation. *QfSns.uia2-6A* was detected in all eight datasets (17-AB, 18-AB, 19-AB, 18-ASH, 18-WW, 18-SS, 19-SS, and BLUP data) and explained 8 to 20% of the phenotypic variation. *QfSns.uia2-7D* was also detected in all eight datasets and explained 17 to 44% of the phenotypic variation. Like GY, HD, and HT QTL on 7DS, the QTL *QfSns.uia2-7D* had the biggest effect on fSNS (Table 2) and was mapped in the flanking region of *FT-D1* (Fig. 2a). Except for *QfSNS.uia2-6A,* positive alleles for all remaining QTL were contributed by LCS Star.

#### QTL for TKW

A total of three QTL were detected for TKW on chromosome 4A, 6A, and 7D (Table 2). *QTkw.uia2-4A* was detected in five of eight datasets (17-AB, 18-AB, 18-MSC, 18-SS, and BLUP data), explaining 8 to 13% of the phenotypic variation. *QTkw.uia2-6A* was detected in three of eight datasets (17-AB, 18-SS, and BLUP data), explaining 11 to 14% of the phenotypic variation. *QTkw.uia2-7D* was detected in three of eight datasets (18-AB, 19-AB, and BLUP data), explaining 17 to 27% of the phenotypic variation. The *QTkw.uia2-7D* was mapped in the flanking region of *FT-D1* (Fig. 2a). Positive alleles for TKW were contributed by LCS Star for *QTkw.uia2-4A* and by UI Platinum for *QTkw.uia2-6A* and *QTkw.uia2-7D*.

#### QTL for PTN

Two QTL were detected for PTN on chromosomes 4A and 6A (Table 2). *QPtn.uia2-4A* was detected in three of five datasets (18-AB, 18-ASH, and BLUP data), explaining 7 to 10% of the phenotypic variation. *QPtn.uia2-6A* was detected in all five datasets (18-AB, 18-ASH, 18-WW, 19-AB, and BLUP data), explaining 7 to 20% of the phenotypic variation. The positive alleles for PTN were contributed by UI Platinum for *QPtn.uia2-4A* and by LCS Star for *QPtn.uia2-6A*.

### QTL cluster on chromosome 7D and saturated map with KASP markers

Five QTL (*QGy.uia2-7D, QHt.uia2-7D*, *QHd.uia2-7D, QfSns.uia2-7D*, and *QTkw.uia2-7D*) were mapped in a flanking region on chromosome 7D, designated herein as the 7D QTL cluster, and the peak SNP marker for all traits was close to the flowering gene *FT-D1* (Fig. 2a and Table 2). The QTL intervals for the five QTL were all mapped from 0 to 16.09 cM and the two flanking markers (*IWB18914* and *IWB40120*) spanned 37.5 Mbp in physical distance. To saturate this region, seven additional KASP markers were designed using the capture data from exome and promoter regions and were mapped in this QTL region (Fig. 2b, Table 2, and Supplemental Table 2). The two flanking KASP markers (*7DS-66* and *7DS-71*) for the QTL in the saturated map spanned only 5.2 Mbp in physical distance (Fig. 2b). With the saturated map, QTL were detected in additional locations: *QGy.uia2-7D* in 19-SS and *QTKW.uia2-7D* in 18-SS (Table 2).

### QTL clusters on chromosomes 6A and 4A

QTL cluster on chromosome 6A includes *QPtn.uia2-6A, QfSns.uia2-6A*, *QTkw.uia2-6A*, and *QHd.uia2-6A* based on the map position in Table 2. The LCS Star allele contributed to more PTN, but less fSNS and TKW, and later heading. QTL intervals of this QTL cluster were large, and therefore, additional populations may be needed to dissect the QTL cluster for each of the three traits.

QTL cluster on 4A includes *QPtn.uia2-4A, QTkw.uia2-4A,* and *QHt.uia2-4A.1* based on the map position in Table 2. The LCS Star allele contributed to greater plant height and larger TKW, and the UI Platinum allele contributed to more PTN. All three QTL had smaller effects compared to the QTL identified in other clusters on 6A and 7D. The LCS Star alleles for greater TKW and HT may be more important than the UI Platinum allele for PTN since QTL for these alleles were detected in more environments.

### QTL validation in the diverse spring wheat line panel

Six KASP markers (Supplemental Table 2) were used in the allelic effect analysis of the diverse spring wheat line panel, which include one (*4A-102*) on chromosome 4A, two (*6A-288* and *6A-454*) on 6A, one (*7BS-76*) on 7B, and two (*7DS-66* and *7DS-71*) on 7D (Table 3). Eight out of the 14 allelic effects of QTL measured were significant at various levels of statistical significance (Table 3).

**Table 3:** QTL validation in a diverse spring wheat line panel

QTL/Marker	Allele	Trait	Mean	Difference^a^	*P* value^b^	Sample Size
*QTL-4A*						
*4A-102*	UIP^c^	HT	36.40	− 5.7	< 0.001	9
	LCS		42.10			161
*4A-102*	UIP	TKW	36.20	1.2	0.24	9
	LCS		35.00			161
*4A-102*	UIP	PTN	447.80	− 2.8	0.38	9
	LCS		450.60			161
*QTL-6A*						
*6A-288*	UIP	HD	134.80	1.21	0.017	89
	LCS		133.59			81
*6A-454*	UIP	fSNS	17.20	0.3	0.05	102
	LCS		16.90			68
*6A-288*	UIP	TKW	37.10	1.5	0.0011	89
	LCS		35.60			81
*6A-288*	UIP	PTN	445.4	− 13.2	< 0.001	89
	LCS		458.6			81
*QTL-7B*						
*7BS-76*	UIP	HD	132.7	− 1.2	0.003	67
	LCS		133.9			93
*7BS-76*	UIP	fSNS	17.22	− 0.79	0.011	67
	LCS		18.01			93
*QTL-7D*						
*7DS-66*	UIP	GY	64.86	− 4.63	< 0.001	62
	LCS		69.49			108
*7DS-66*	UIP	HT	41.40	− 0.6	0.39	62
	LCS		42.00			108
*7DS-66*	UIP	HD	133.80	− 1.2	< 0.001	62
	LCS		135.00			108
*7DS-71*	UIP	fSNS	17.00	− 0.12	0.73	50
	LCS		17.12			120
*7DS-66*	UIP	TKW	36.09	− 0.05	0.91	62
	LCS		36.14			108

^a^The difference is calculated using the mean of the entries with the UIP allele minus the mean of the entries with the LCS allele

^b^Significance in the *T* test for the two allele groups

^c^UIP or LCS stands for the alternative allele for a peak SNP or KASP associated with the specific QTL corresponding to Table 2

### Additive effect of the two QTL on 6A and 7D for TKW, fSNS, and HD

QTL for fSNS and TKW were detected on chromosomes 6A and 7D in multiple environments (Table 2). UI Platinum contributed favorable alleles for both QTL on the chromosomes 6A and 7D for TKW, and the two QTL had an additive effect toward increasing TKW (Table 4). The fSNS QTL on chromosome 6A and on 7D also had an additive effect, but the favorable alleles of the two QTL were from different parents, the allele of 7D QTL from LCS Star has a greater effect than the allele of 6A QTL from UI Platinum (Table 4). The allelic effects of the two QTL for HD worked in the same way as the fSNS QTL allelic effects (Table 4).

**Table 4:** Additive effects of the QTL on 6A and 7D for TKW, fSNS, and HD in the UI Platinum x LCS Star-derived doubled haploid population*

Haplotype^a^	TKW	fSNS	HD
AABB^b^	35.3A^c^	18.3A	174.7A
aaBB	34.1AB	17.6B	174.2B
AAbb	33.5B	17.2C	173.1C
aabb	32.4C	16.6D	172.7C

^*^The number of lines used in each haplotype AABB, aaBB, AAbb, and aabb were different, which are 39:55:27:60 for TKW, 57:35:57:31 for fSNS, 58:38:57:28, respectively

^a^The markers listed in Supplemental Table 2 were used to build the haplotypes

^b^A/a indicates the 6A QTL, while B/b indicates the 7D QTL. Capital letters indicate alleles that increase trait values and small letters indicate alleles that decrease trait values

^c^All pair means were compared using the Tukey–Kramer HSD method. Values followed by the same capital letter are not significantly different at p 0.05

## Discussion

The present study used a DH population derived from two high-yielding spring wheat cultivars to dissect the genetic basis of variation for GY, three major yield components (fSNS, PTN, and TKW), two agronomic traits HD and HT. By combining phenotyping in eight field trials and genotyping with the high-density genotyping platform, we identified major QTL controlling these traits in spring wheat. Of the 19 QTL detected, 14 were clustered in four genomic regions on chromosomes 4A (three QTL), 6A (four QTL), 7B (two QTL), and 7D (five QTL). KASP markers for the four QTL clusters were developed and assessed in an elite spring wheat panel of 170 lines, eight of the 14 QTL were verified. These KASP markers can be used in the primary selection of the three yield component traits and GY.

### QTL analysis and comparison with previous studies

The present study confirmed QTL for PTN on chromosomes 4A and 6A, which were identified in previous work using a population from UI Platinum x SY Capstone (Wang et al. 2018). In both studies, UI Platinum had the 4A allele for higher PTN and the 6A allele for lower PTN. QTL for TKW on chromosomes 4A and 6A, and QTL on 7BS and 7DS for GY, fSNS, TKW, and HD were likely not detected in the previous study because UI Platinum and SY Capstone showed little divergence in these traits.

The present study identified a QTL for GY on chromosome 7D (*QGY.uia2-7D*) (Table 2). Groos et al. (2003) and Narasimhamoorthy et al. (2006) previously reported three grain yield QTL on 7D using SSR, RFLP, and AFLP markers. As summarized in Zhang et al. (2018), these three QTL were located near the centromere or at the long arm of the chromosome, which differs from the position of *QGy.uia2-7D* identified in the present study and thus supports the hypothesis that *QGy.uia2-7D* is novel.

Four QTL were identified for HD on chromosomes 4B, 6A, 7B, and 7D (Table 2). *QHd.uia2-4B* and *QHd.uia2-6A* have not been previously reported, which suggests these two QTL could be novel. *QHd.uia2-7B* and *QHd.uia2-7D* were both mapped on the short arms of chromosomes 7B and 7D. QTL at similar locations were previously reported and the QTL for HD showed either limited or no effects on GY (Maccaferri et al. 2008 and Zhang et al. 2018). In the present study, the QTL for HD on 7DS was mapped in the flanking region for the QTL of GY. Lines with later maturity tend to have higher GY (Table 1).

There were five QTL detected for HT on chromosomes 4A (2), 5A, 5D, and 7D (Table 2). *QHt.uia2-4A.1* spanned both the short arm and long arm of chromosome 4A while *QHt.uia2-4A.2* was mapped on the long arm of chromosome 4A. *QHt.uia2-5A* was mapped on the long arm of chromosome 5A, and *QHt.uia2-5D* was mapped on the short arm of chromosome 5D. Lastly, *QHt.uia2-7D* was mapped on the short arm of chromosome 7D. Of the five QTL detected for HT, three have not been previously reported (*QHt.uia2-4A.1, QHt.uia2-4A.2,* and *QHt.uia2-7D*) and therefore could be novel.

Four QTL for fSNS were identified on chromosomes 5D, 6A, 7B, and 7D. *QfSns.uia2-5D* was mapped on the long arm of chromosome 5D, and this locus could correspond to the chromosome 5D QTL for fSNS identified by Li et al. (2007). *QfSns.uia2-6A* occurred in a large region spanning both the short and long arms of chromosome 6A (Table 2). *QfSns.uia2-6A* could possibly correspond to the QTL associated with fSNS on the short arm of chromosome 6A, which was previously identified by Kumar et al. (2007) and Wang et al. (2011). *QfSns.uia2-7B* was mapped on the short arm of chromosome 7B and *QfSns.uia2-7D* was detected on the short arm of chromosome 7D (Fig. 2a). Wang et al. (2011) reported QTL associated with fSNS on the long arms of both chromosomes 7B and 7D. Since no QTL for fSNS were previously identified on the short arms of these two chromosomes, *QfSns.uia2-7B* and *QfSns.uia2-7D* could be novel. The effects of the two QTL could be possibly the pleiotrophic effect of *FT-B1* and *FT-D1*. Later flowering favored the more fSNS as supported by the positive correlation between HD and fSNS (Table 1).

QTL for TKW were detected on chromosomes 4A, 6A, and 7D. *QTkw.uia2-4A* was mapped in a large region spanning both the long and short arms of chromosome 4A (Fig. 2). Gao et al. (2015) detected a QTL for TKW on the long arm of chromosome 4A, and perhaps *QTkw.uia2-4A* corresponds to the QTL they found. *QTkw.uia2-6A* was mapped to a region spanning both the short and long arms of chromosome 6A (Table 2). Li et al. (2007) and Gao et al. (2015) also detected a QTL on chromosome 6A in the same region. In addition, the *TaGW2-A* gene is located at the 237 Mbp locus (Zhai et al. 2018), which is 36 Mbp distant from the *QTkw.uia2-6A* (201 Mbp in Table 2)*. QTkw.uia2-7D* was mapped on the short arm of chromosome 7D (Table 2 and Fig. 2a and Fig. 2b).

QTL for PTN were detected on chromosomes 4A and 6A. *QPtn.uia2-4A* was mapped in a large region spanning both the short and long arms of chromosome 4A (Fig. 2). Wang et al. (2018) also reported QTL associated with PTN on chromosome 4A. *QPtn.uia2-6A* was mapped in a large region spanning both the short and long arms of chromosome 6A (Fig. 2). Sukumaran et al. (2015) reported a linkage block in this region of approximately 77–81 cM that encompassed 63% of the entire 6A chromosome (100–500 Mbp). Our results showed the QTL on chromosome 6A was located at 90–530 Mbp with no obvious peaks. Wang et al. (2018) used a DH population with a common parent (UI Platinum) and found a QTL for PTN in the same region contributed by the SY Capstone parent, which supports the hypothesis that LCS and SY Capstone contain the same allele for PTN on chromosome 6A.

### QTL in relation to *FT-B1* and *FT-D1*

Three important genes, known as vernalization (*Vrn*), photoperiod (*Ppd*), and *FLOWERING LOCUS T* (*FT*), have been associated with spikelet number per spike (SNS) and HD. *VRN1* is one of the major genes controlling HD in winter wheat (Fu et al. 2005; Yan et al. 2003). Li et al. (2019) demonstrated that *vrn1*-null mutants have increased SNS because of a longer transition time from inflorescence meristem to a terminal spikelet. Ochagavia et al. (2018) reported that a photoperiod-insensitive allele (*Ppd-1a*) influences SNS. Dixon et al. (2018) and Finnegan et al. (2018) demonstrated that deletion of *FT-B1* delays the transition to reproductive growth, increasing spikelet number. Most recently, Chen et al. (2020) showed that *FT-A1* has pleiotropic effects on spikelet number and heading date. Liu et al. (2020) showed that *FT-D1* has pleiotropic effects on thousand kernel weight. The QTL for fSNS located in the flanking regions of *FT-B1* identified in the present study supports these previous findings by Dixon et al. (2018) and Finnegan et al. (2018).

The present study is the first to demonstrate the colocalization of QTL for GY, fSNS, TKW, HD, and *FT-D1*. UI Platinum contributed to higher TKW, while LCS Star contributed more to fSNS, a later HD, a later flowering time, and higher GY. Based on the positions of these QTL and the correlations among GY, fSNS, HD, and TKW, the effect of these QTL may result either from different closely linked genes or from the pleiotropic effects of *FT-D1.* Future work to dissect the 7DS QTL cluster could help determine whether *FT-D1* has pleiotropic effects on the four traits.

### Prospects and challenges

A total of 19 QTL were detected on seven wheat chromosomes for four yield-related traits (GY, fSNS, TKW, and PTN) and two agronomic traits (HD and HT). Of the 19 QTL, favorable alleles for 11 QTL were from LCS Star and eight were from UI Platinum. This result suggests that pyramiding favorable alleles from both parents may increase the values of each yield component and GY. The present study developed KASP markers for QTL associated with the three yield component traits and GY (Supplemental Table 2). These KASP markers will be used for fine mapping and to dissect the four QTL clusters and, ultimately, to clone the three QTL for yield component traits.

A challenge posed from results of our study is how to select genetic improvement of related yield component traits that have negative trade-offs. Four chromosomes, 4A, 6A, 7B, and 7D, each contained multiple QTL within which the associated traits were negatively correlated. For example, selecting the LCS Star allele for fSNS on chromosome 7D will result in selecting smaller TKW and selecting the UI Platinum allele for TKW on chromosome 6A will result in selecting less PTN. In practical breeding, it is a great challenge to balance PTN, TKW, and fSNS. Molecular-level results from the present study explain the complexity of this challenge and may make it possible to break the linkage among the three traits via molecular marker-assisted selection and gene cloning.

For the QTL region on chromosome 7D, considering the number of trials that a QTL was detected, percentage of phenotypic variation explained by the QTL, and the broad-sense heritability of the trait, the QTL *QfSns.uia2-7D* is more important than the QTL *QTkw.uia2-7D*. Therefore, selecting the LCS Star allele for higher fSNS may be more effective than selecting the higher TKW allele from UI Platinum.

The QTL cluster on 7BS consisted of two QTL, *QHd.uia2-7BS* and *QfSns.uia2-7B*, and the LCS Star allele contributed to more fSNS and later heading. This QTL cluster was in the flanking region of *FT-B1.* Although the effect of the LCS Star allele at *QfSns.uia2-7BS* was much smaller than that of *QfSns.uia2-7DS,* the combined effect of the two QTL may contribute to higher yield.

To balance PTN, TKW, and fSNS for a specific production environment, we propose four selection schemes for the 6A and 7DS QTL clusters. Selecting the LCS Star alleles for PTN at the QTL 6A cluster and fSNS alleles at the QTL 7DS cluster should result in more PTN and fSNS, but lower TKW and later heading. This plant architecture may be good in environments that favor tillering, such as irrigated (18-AB, 18-ASH, and 19-AB) and high rainfall rainfed areas (18-WW).

Selecting UI Platinum alleles at the two QTL clusters should result in less PTN and fSNS, but higher TKW. This architecture may be best in environments that have longer grain filling time to produce larger TKW for higher grain yield.

Selecting UI Platinum alleles for fSNS and TKW/PTN at the QTL 6A cluster and the UI Platinum allele for fSNS at the QTL 7DS cluster should result in less PTN but more fSNS and higher TKW. To enable this selection scheme, additional research will be needed to understand the relationship (linkage or pleiotropy) of the three QTL (*QPtn.uia2-6A, QfSns.uia2-6A*, and *QTkw.uia2-6A*) in environments that favor fast or longer grain filling time.

A final selection scheme would be the LCS Star allele for PTN at the QTL 6A cluster combined with the UI Platinum allele for TKW at the 7DS QTL cluster. To achieve this selection scheme, additional research is essential to understand the relationship (linkage or pleiotropy) of the five QTL *QTkw.uia2-7DS, QfSns.uia2-7DS*, *QGy.uia2-7DS, QHd.uia2-7DS,* and *QHt.uia2-7DS* in environments that favor either shorter or longer grain filling time.

## Supplementary information

Supplementary file1 (DOCX 23kb)
