# Supplementary material for: QTL mapping for grain yield and three yield components in a population derived from two high-yielding spring wheat cultivars
Source: Theor Appl Genet. 2021 Mar 9;134(7):2079–95. doi: 10.1007/s00122-021-03806-1 (PMC8263538; doi:10.1007/s00122-021-03806-1)
Supplement: Supplementary file 1 — Supplementary file1 (DOCX 23kb) [file 122_2021_3806_MOESM1_ESM.docx]

**Supplemental Table 1.** Summary of linkage groups derived from linkage analysis with 90K SNP markers in the UI Platinum x LCS Star derived doubled haploid population.

| **Chromosome** | **No. of Linkage Groups** | **No. of SNP** | **Length**  **(cM)** | **Marker Density (marker/cM)** |
| --- | --- | --- | --- | --- |
| 1A | 2 | 55 | 159.90 | 0.34 |
| 2A | 2 | 105 | 283.51 | 0.37 |
| 3A | 3 | 66 | 111.68 | 0.59 |
| 4A | 2 | 50 | 107.02 | 0.47 |
| 5A | 3 | 69 | 162.68 | 0.42 |
| 6A | 1 | 58 | 101.57 | 0.57 |
| 7A | 4 | 86 | 396.10 | 0.22 |
| 1B | 3 | 61 | 118.23 | 0.52 |
| 2B | 1 | 113 | 229.24 | 0.49 |
| 3B | 2 | 81 | 220.88 | 0.37 |
| 4B | 2 | 62 | 179.99 | 0.34 |
| 5B | 2 | 111 | 248.30 | 0.45 |
| 6B | 2 | 62 | 289.14 | 0.21 |
| 7B | 3 | 93 | 260.23 | 0.36 |
| 1D | 1 | 35 | 111.36 | 0.31 |
| 2D | 2 | 45 | 260.29 | 0.17 |
| 3D | 3 | 32 | 155.55 | 0.21 |
| 4D | 2 | 14 | 94.24 | 0.15 |
| 5D | 3 | 31 | 213.37 | 0.15 |
| 6D | 3 | 22 | 48.47 | 0.45 |
| 7D | 2 | 25 | 141.06 | 0.18 |
| A genome | 17 | 489 | 1322.46 | 0.37 |
| B genome | 15 | 583 | 1546.01 | 0.38 |
| D genome | 16 | 204 | 1024.34 | 0.20 |
| Total | 48 | 1276 | 3892.81 | 0.33 |

**Supplemental Table 2.** KASP markers developed for QTLs associated with grain yield (GY), heading date (HD), plant height (HT), thousand kernel weight (TKW), fertile spikelet number per spike (fSNS), and productive tiller number per unit area (PTN) in the UI Platinum x LCS Star derived doubled haploid population.

| *Trait* | KASP | SNP Name | Position** (bp) | Primer Type | Primer Sequence (5’ – 3’) |
| --- | --- | --- | --- | --- | --- |
| *HT/TKW/PTN* | *4A-102** | *IWB33052* | 102614749 | FAM | gaaggtgaccaagttcatgctctgaacaaaaccagtagctagttac |
|  |  |  |  | HEX | gaaggtcggagtcaacggattctgaacaaaaccagtagctagttaa |
|  |  |  |  | Common | tttgacttcctgagcatctactat |
| *HD/TKW/PTN* | *6A-288** | *IWB10321* | 288411565 | FAM | gaaggtgaccaagttcatgcttggggctcaccttgaatcc |
|  |  |  |  | HEX | gaaggtcggagtcaacggatttggggctcaccttgaatct |
|  |  |  |  | Common | gccagctataatatgcctcaaatat |
| *fSNS* | *6A-454** | *IWB35333* | 454649338 | FAM | gaaggtgaccaagttcatgctgctttcatctgttgttgtgattcag |
|  |  |  |  | HEX | gaaggtcggagtcaacggattgctttcatctgttgttgtgattcat |
|  |  |  |  | Common | gctctgattcgaccagtgct |
| *HD/fSNS* | *7BS-76** | *IWB76332* | TraesCS7B01G013100 | FAM | gaaggtgaccaagttcatgctaatggccggtagggataggg |
|  |  |  |  | HEX | gaaggtcggagtcaacggattaatggccggtagggataggc |
|  |  |  |  | Common | gaggttggtggtccggacg |
| *GY/HD/HT/TKW/fSNS* | *7DS-11* | *KS0617_916184* | 11636416 | FAM | gaaggtgaccaagttcatgctcctgaatagccttgcccct |
|  |  |  |  | HEX | gaaggtcggagtcaacggattcctgaatagccttgccccg |
|  |  |  |  | Common | atcgttgcacaagttgatgg |
| *GY/HD/HT/TKW/fSNS* | *7DS-42* | *KS0617_921518* | 42074348 | FAM | gaaggtgaccaagttcatgctcttgaggacaacaattatgtgacg |
|  |  |  |  | HEX | gaaggtcggagtcaacggattcttgaggacaacaattatgtgaca |
|  |  |  |  | Common | tgttcgaatcactgggtatcatt |
| *GY/HD/HT/TKW/fSNS* | *7DS-51* | *KS0617_923024* | 51952308 | FAM | gaaggtgaccaagttcatgctcacctttaatttggttgatctcca |
|  |  |  |  | HEX | gaaggtcggagtcaacggattcacctttaatttggttgatctccg |
|  |  |  |  | Common | gttcttctccatgtcctgcat |
| *GY/HD/HT/TKW/fSNS* | *7DS-59* | *KS0617_924690* | 59737774 | FAM | gaaggtgaccaagttcatgctacccaagaacctacgacgtc |
|  |  |  |  | HEX | gaaggtcggagtcaacggattacccaagaacctacgacgtt |
|  |  |  |  | Common | gctccattcggatcctgtga |
| *GY/HD/HT/TKW/fSNS* | *7DS-62* | *KS0617_925111* | 62214499 | FAM | gaaggtgaccaagttcatgctgcttagcactgcacactccg |
|  |  |  |  | HEX | gaaggtcggagtcaacggattgcttagcactgcacactcca |
|  |  |  |  | Common | gtatcccctactgcatacttgac |
| *GY/HD/HT/TKW* | *7DS-66** | *KS0617_925860* | 66074035 | FAM | gaaggtgaccaagttcatgctatgcgcccaattttcatcct |
|  |  |  |  | HEX | gaaggtcggagtcaacggattatgcgcccaattttcatccc |
|  |  |  |  | Common | cctggagctcagtctgcc |
| *fSNS* | *7DS-71** | *KS1217_209987* | 71342971 | FAM | gaaggtgaccaagttcatgctcatgaggaacttgaggagagg |
|  |  |  |  | HEX | gaaggtcggagtcaacggattcatgaggaacttgaggagagc |
|  |  |  |  | Common | gcgcgcacagatgggttt |

* Markers used in allelic analysis of the diverse spring wheat line panel. ** SNP positions based on Chinese RefSeq v1.0.
